# Selective dual-band metamaterial perfect absorber for infrared stealth technology

**DOI:** 10.1038/s41598-017-06749-0

**Published:** 2017-07-27

**Authors:** Jagyeong Kim, Kiwook Han, Jae W. Hahn

**Affiliations:** Nano Photonics Laboratory, School of Mechanical Engineering, Yonsei University, 50 Yonsei-ro, Seodeamun-gu, Seoul, 03722 Republic of Korea

## Abstract

We propose a dual-band metamaterial perfect absorber with a metal–insulator–metal structure (MIM) for use in infrared (IR) stealth technology. We designed the MIM structure to have surface plasmon polariton (SPP) and magnetic polariton (MP) resonance peaks at 1.54 μm and 6.2 μm, respectively. One peak suppresses the scattering signals used by laser-guided missiles, and the other matches the atmospheric absorption band, thereby enabling the suppression of long-wavelength IR (LWIR) and mid-wavelength IR (MWIR) signals from objects as they propagate through the air. We analysed the spectral properties of the resonance peaks by comparing the wavelength of the MP peak calculated using the finite-difference time-domain method with that obtained by utilizing an inductor–capacitor circuit model. We evaluated the dependence of the performance of the dual-band metamaterial perfect absorber on the incident angle of light at the surface. The proposed absorber was able to reduce the scattering of 1.54 μm IR laser light by more than 90% and suppress the MWIR and LWIR signatures by more than 92%, as well as maintain MWIR and LWIR signal reduction rates greater than 90% across a wide temperature range from room temperature to 500 °C.

## Introduction

Metamaterial perfect absorbers have attracted considerable attention in energy-harvesting applications such as thermophotovoltaics^1–4^, LED-light-extracting structures^5^, spectroscopic sensors^6^, and near-field lithography^7^. Metal–insulator–metal (MIM)^8–10^ and photonic crystal^11–13^ structures are the most popular configurations for selective absorbers. However, the low peak emissivities of photonic crystal structures limit the spectral properties of absorbers with such designs^1, 13^. On the other hand, MIM structures exhibit exotic properties in terms of absorption and wavelength selectivity^1, 8, 14–18^. Owing to the plasmonic phenomenon that occurs at the metal–dielectric interfaces, the absorption nearly reaches unity at certain wavelengths, enabling the realization of perfect absorbers. Various array structures including T-shaped plasmonic multilayers^19^, split-ring resonators^20^, cross sub-lattices^10^, and square patches^21^ are applied in MIM structures.

Stealth technology is vital in the military, because it facilitates the acquisition of control over strategically important areas and the destruction of key targets to ensure survival and to enable invasions. In general, stealth technology involves the achievement of low observability by reducing signal detection or resending countermeasure signals. The detector observes infrared, radar, and acoustic signals reflected by or emitted from the targets^22, 23^. Therefore, researchers have endeavoured to reduce the scattering and reflection of radar waves from the surfaces of objects that could be detected by radar detection systems^24, 25^.

Research on the development of frequency-selective devices using MIM structures for stealth technology applications^24–28^ has been very popular. Many studies have been conducted on metamaterial perfect absorbers to improve the performance of stealth technology using radar-absorbing surfaces. These studies have also been extended to include symmetric MIM structures such as composite unit cells^1, 28^, stacked layers^29, 30^, and multiple rings^18, 31^ to achieve a performance that is insensitive to variations in polarization and incident angles. Owing to its property of strong absorption of electromagnetic waves, the research on stealth technology using MIM structures has mostly focused on reducing the cross section of electromagnetic waves in the frequency range from GHz to THz.

Since the IR perfect absorber using an MIM structure increases the IR signature of target objects by enhancing their thermal radiation, the applications of perfect absorbers in IR stealth technology have not been generally accepted. In previous studies, IR perfect absorbers were developed for the application to IR radiation sources rather than to IR stealth technology^1–4^. As an alternative, they adjusted the spectral band of the perfect absorber to suppress the IR signature^31^. Currently, IR technology is commonly applied in thermal imaging devices for surveillance systems, which measure the thermal radiation of objects^22^, as well as in laser-guided missiles to improve the accuracy of attacks on military targets by detecting scattered IR laser light to guide missiles to their targets^23^. The thermal radiation emitted by an object provides the main IR signature that is used for thermal imaging by IR tracking devices, and depends on the temperature, size, and shape of the object. In addition, IR waves scattered from target surfaces provide important IR signatures that are utilized by laser-guided missiles. The wavelength of the IR signature depends on the type of IR detector that is utilized, and can be between 2 μm and 14 μm. Since the IR signature of an object depends on various parameters related to its size and surface properties, such as its temperature, emissivity, and reflection geometry, there are various methods of reducing IR signatures to achieve IR stealth technology^32^. Decreasing the surface temperature to suppress the thermal radiation from the target surface is the most popular approach. In addition, exhaust plume shielding is adopted to change the sizes and shapes of IR sources. IR homing technology has been developed following the progress in IR detector technology in terms of sensitivity and resolution. IR search and track (IRST) systems detect thermal radiation from the surfaces of military vehicles and combustion exhaust for the purpose of surveillance and to guide missiles to their targets.

Surface property modifications have also been studied to enable the suppression of IR signatures of objects and the development of IR stealth technology^31, 33–35^. Metal-powder–resin coating^33^ was reported to yield low surface emissivity in the long-wavelength IR (LWIR) range of 8–14 μm. To achieve low observability, the contrast radiant intensity was reduced by covering the surface of a tank with tiles, whose temperature was held at ambient temperature to make the tank IR-invisible^36^. Meanwhile, frequency-selective devices with T-shaped MIM structures and silver (Ag) nanoparticles have been reported to attenuate the IR signatures of vehicles, in the atmosphere^34, 35^.

In the present work, we propose a dual-band metamaterial perfect absorber with an MIM structure for use in IR stealth technology. It is a perfect absorber at a wavelength of 1.54 μm and can thereby reduce the IR signatures used by laser-guided missiles. We designed the absorption peak bandwidth to be extremely narrow to minimize the thermal radiation at 1.54 μm. The perfect absorber also has a broad absorption peak that matches the atmospheric absorption band, and enables the attenuation of IR signatures as they propagate through the air. To address the practical usability of the proposed absorber, we analysed the dependence of its spectral characteristics on the polarization and angle of the incident laser beam.

## MIM structure design for IR stealth technology

### Narrow-band near-IR metamaterial perfect absorber

First, we designed the MIM structure of the metamaterial perfect absorber to match the wavelength of a 1.54 μm laser beam. This structure reduces the scattering of IR laser beams from surfaces, thereby suppressing the IR signatures that would guide laser-guided missiles to their targets. However, to avoid enhancing the IR signature in the short-wavelength IR (SWIR) range of 1–3 μm, it was necessary to make the spectral bandwidth of the metamaterial perfect absorber as narrow as possible. The array consists of nanoscale Ag disks on a substrate containing dielectric and metal layers to form an MIM structure. This structure causes multiple Bloch-mode surface plasmon polariton(SPP)s to interfere constructively, thus creating a very sharp spectral peak^36–38^. The dependence of the spectrum on the polarization of the incident wave can be suppressed by using symmetric disks^39^.

**Figure 1:**
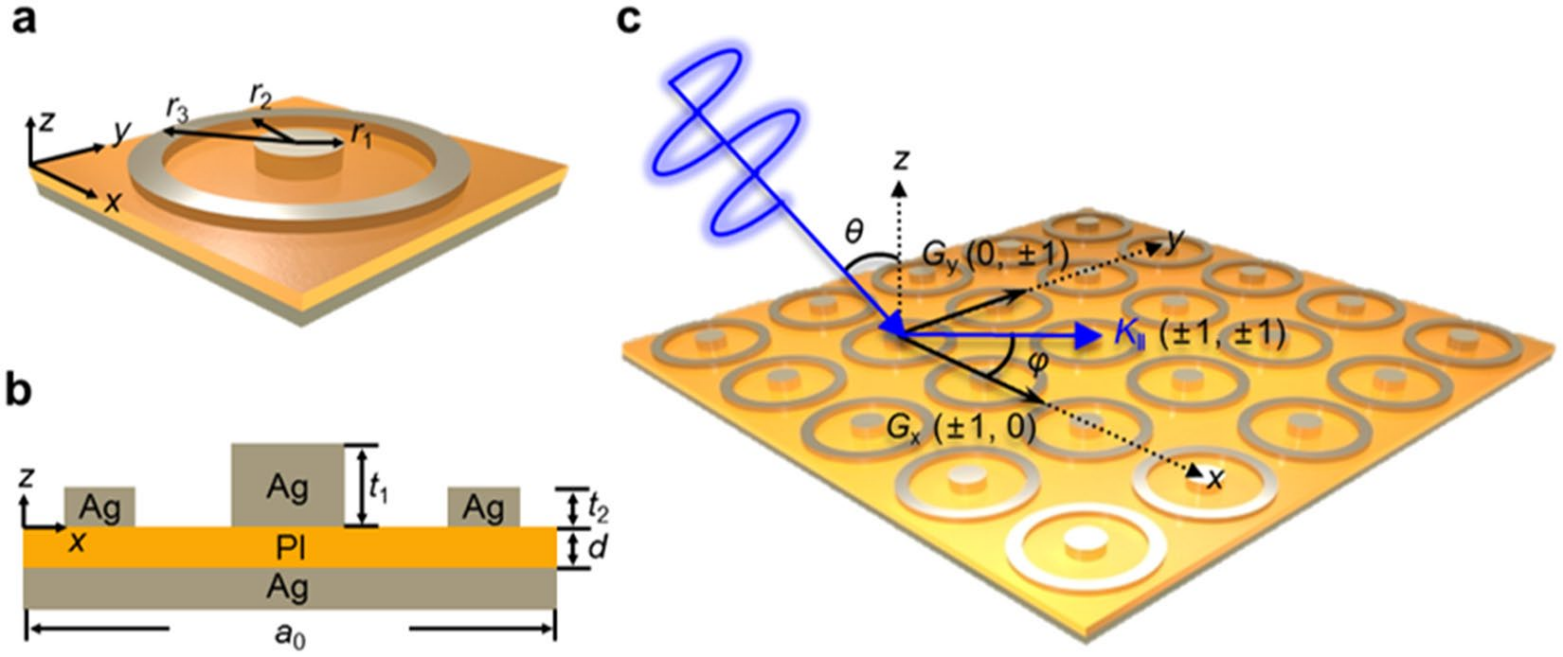
Schematic diagram and surface plasmon polariton (SPP) excitation of the dual-band perfect absorber. (**a**) Unit cell of the circular ring–disk structure, where the disk radius is *r*_1_ and the inner and outer radii of the ring are *r*_2_ and *r*_3_, respectively. (**b**) Cross-sectional view of the MIM structure composed of Ag and polyimide (PI) layers having thicknesses of *t*_1_, *t*_2_, and *d*, and a unit cell period of *a*_0_. (**c**) Incident light (red line) with polar angle *θ* encounters the periodic pattern. The first Brillouin zone is defined by connecting the perpendicular bisectors of the reciprocal lattice, and a portion of the incident light propagates in the plane containing the periodic pattern with azimuthal angle *φ*.

We determined the period *a*_0_ of the disks in the array by considering the plasmonic effects needed to excite SPPs with a sharp resonance peak at 1.54 μm, which is the wavelength of a near-IR laser. The wavelength, magnitude, and width of the resonance peak depend on the distance between the disks in the array, the angle and polarization of the incident light, and material properties^37^. Figure 1(c) depicts a 2D array consisting of circular metal disks and rings. When incident light encounters the array surface, a portion of the light on the surface acts like a Bloch wave, whose wave function is that of a particle in a periodic potential^36, 37^. The periodicity contributes to the incident light by adding to its momentum. In this structure, the unit cells are symmetric in the x and y directions; therefore, |*G*_x_| = |*G*_y_| = 2π/*a*_0_, where *G*_x_ and *G*_y_ are reciprocal vectors that are inversely proportional to the grating period. If a certain momentum, caused by the periodicity, is applied to the incident light, an SPP Bloch wave appears with *k*_‖_ + *iG*_x_ + *jG*_y_ = *k*_spp_, where the indices *i* and *j* represent the direction of the dominant propagation vector in the 2D periodic array. Here, (*i*, *j*) = (0, ±1), (±1, 0), or (±1, ±1) in the first Brillouin zone, *k*_‖_ is the plane wave of the incident light as a function of *θ* and *ϕ*, and *k*_spp_ is the dispersion relation of SPPs on a metal–dielectric interface that has a greater momentum than that of the incident light. By assuming that the light is normally incident and has transverse magnetic (TM) polarization, the equation for *a*_0_ for an SPP Bloch wave can be expressed as

1 $${a}_{0}={\lambda }_{{\rm{s}}{\rm{p}}{\rm{p}}}\sqrt{{i}^{2}+{j}^{2}}\sqrt{\frac{{\varepsilon }_{{\rm{P}}{\rm{I}}}+{\varepsilon }_{{\rm{A}}{\rm{g}}}}{{\varepsilon }_{{\rm{P}}{\rm{I}}}{\varepsilon }_{{\rm{A}}{\rm{g}}}}}$$

where *λ*_spp_ = 1.54 μm and the relative permittivities of the dielectric and metal are 2.56  and −85.5 , respectively^40, 41^. Momentum is applied to the 1.54 μm incident light with a propagation direction of (±1, ±1), when *a*_0_ = 1.34 μm.

### Magnetic polariton excitation by disk-and-ring array to realize a dual-band perfect absorber

The spectral properties of SPPs, such as resonance wavelength and peak height, depend on the polarization of the laser beam. As shown in Fig. 1(a), we employed rotationally symmetric combinations of disks and rings to prevent the spectral properties from changing. It was also necessary to account for the dependence of the spectral properties on the incident angle indicated in Fig. 1(c).

To retain the IR stealth functionality of the absorber at various incident angles, the SPP resonance peak needed to be kept exactly at the wavelength of the laser beam (1.54 μm). In addition to SPPs, the magnetic polaritons (MPs), which arise from the coupling between external electromagnetic fields and magnetic resonances inside structures, can be excited by laser beams in MIM structures^14–16, 36^ and are well known to be insensitive to the angle of the incident light^36^. Since each unit cell of the MIM structure depicted in Fig. 1 consists of a disk and a ring, two MP resonance peaks corresponding to those two components can be expected. To prevent the SPP Bloch wave resonance peak from shifting away from 1.54 μm due to incident angle variations, the resonance peak generated by the MP of the disk was made coincident with that generated by the SPP of the disk. The wavelength of the MP resonance peak was found to be dependent on the size of the disk. The MP resonance conditions in the MIM structure were analysed using an inductor–capacitor circuit model (ICCM) (see the Supplementary Information). The dimensions of the MIM structure were set such that the resonance peak would occur at the target wavelength and the total impedance of the MIM structure would become zero.

To realize a dual-band perfect absorber for use in IR stealth technology, we designed the MIM structure to yield an additional resonance peak in the mid-wavelength IR (MWIR) range, which is within the strong absorption band of water in the atmosphere. The MPs are excited by the rings shown in Fig. 1, and have a broadband resonance peak that enables low observability by detectors that measure the thermal radiation of objects. Due to Kirchhoff’s law of thermal radiation, absorptivity equals emissivity^1, 4^. In this study, we analysed the resonance conditions of the MPs excited by the rings using an ICCM that was modified by adding inductors in parallel (see the details in the Supplementary Information). The dimensions of the MIM structure were determined using the ICCM and the results of a finite-difference time-domain (FDTD) simulation.

The dual-band metamaterial perfect absorber, depicted in Fig. 1, was designed to have MP resonance peaks at 1.54 μm and 6.2 μm, which differ by almost 5 μm. These two peaks are separately excited by the disks and the rings without interference^42^. To achieve incident angle insensitivity at the laser wavelength of 1.54 μm, the SPP and MP peaks are simultaneously matched to 1.54 μm. The MP peak excited by the rings is a broadband peak and matches the absorption band of water in the atmosphere.

## Results

### Dimensions of the MIM structure

**Figure 2:**
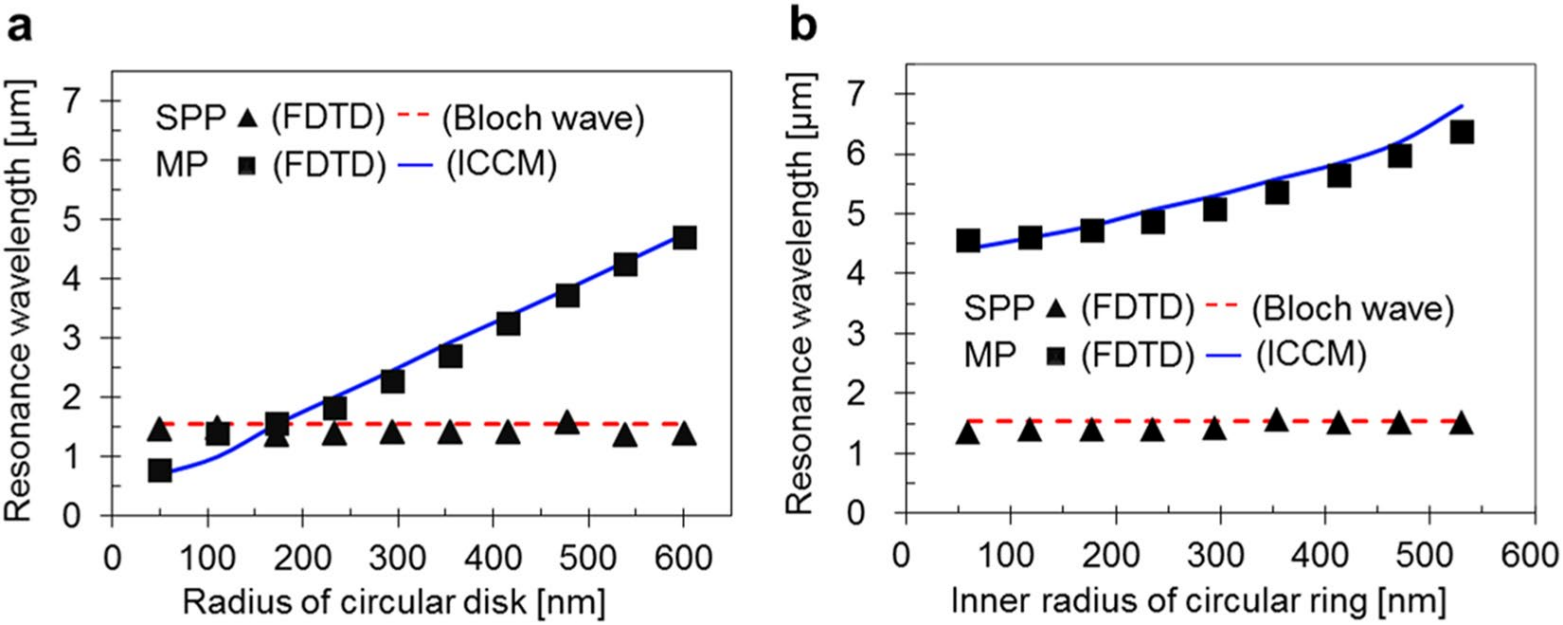
Simulation results and MP and SPP resonance wavelength predictions. Resonance wavelengths of the SPPs and MPs excited by (**a**) the disk array for various disk radii and (**b**) the ring array for various inner radii of the rings. In both the graphs, the Bloch wave and ICCM results (represented by the dashed and solid lines, respectively) correspond closely to the SPP and MP FDTD results (represented by ▲ and ■, respectively).

We determined the dimensions of the MIM structure illustrated in Fig. 1 by using the ICCM and the results of the FDTD simulation, and by calculating the wavelengths of the SPP and MP resonance peaks. The wavelengths of the SPP and MP peaks are plotted in Fig. 2(a) and (b) for various disk radii and inner radii of the rings, respectively. The ICCM results correspond closely to the FDTD results throughout the wide wavelength range of interest.

The resonance wavelength of the SPP Bloch waves depends on *a*_0_, which was set to 1.34 μm to deal with the scattering of the 1.54 μm laser light from the surface and to meet the requirements of the IR stealth technology. Owing to the multiple interference of the SPP waves in the unit cell array^36^, the resonance peak is narrow and its wavelength is insensitive to disk size variations. As seen in Fig. 2(a), the wavelength of the MP resonance peak monotonically increases with the size of the disk. The MP peak curve intersects the SPP Bloch wave curve at a disk radius of 0.168 μm. We set the disk diameter such that the MP resonance wavelength would be identical to that of the SPP Bloch waves, since the MP resonance wavelength is insensitive to the incident angle of the IR laser light. Accordingly, we obtained the dimensions of the MIM structure, the disk radius, and *a*_0_. In the calculations, the top metal layer was 0.18-μm-thick Ag and the insulator layer was PI with a thickness d of 0.1 μm. Note that the spectral properties of the MIM structures are not affected by the metal substrate thickness, when it is greater than 0.1 μm.

To realize a dual-band perfect absorber, we employed unit cells, each of which consisted of a disk and a ring. The spectral properties of the SPPs and MPs for the disk and ring were found to be independent of each other. Therefore, we calculated the spectral properties of each component and combined them to represent the properties of the unit cells. In addition, we introduced an ICCM for the ring in the MIM structure by slightly modifying it for a disk to predict the spectral properties of the rings (see the Supplementary Information). The resonance wavelength of the MPs excited by the rings was predicted using the ICCM and compared with that obtained based on the FDTD simulation results shown in Fig. 2(b). The MP peak excited by the current flow in each individual ring is a broadband peak and lies in the MWIR range, which is necessary to reduce the body temperatures of the objects.

Considering the fabrication tolerance, we allowed the gaps between the unit cells to be larger than 100 nm, which enabled the outer radii of the rings to be 600 nm. Using the ICCM, we calculated the wavelength of the MP peak excited by the rings for various inner radii of the rings, and the results were compared with those obtained using the FDTD, which is shown in Fig. 2(b). The wavelength of the MP peak appears between 4.5 μm and 6.5 μm for inner radii of up to 520 nm. We determined that an inner radius of 500 nm would cause the MP peak wavelength to be 6.2 μm, which is within a strong and broad atmospheric absorption band in the MWIR range. In the calculations, the thickness of each circular ring plate was 100 nm, which was less than that of the disks. As the inner radius of the rings increased with a fixed outer radius of 600 nm, the mutual capacitance (*C*_m2_ in Eq. (S2)), which was proportional to the lower area of the rings, decreased.

### FDTD simulation of field distributions at SPP Bloch wave and MP resonance wavelengths

**Figure 3:**
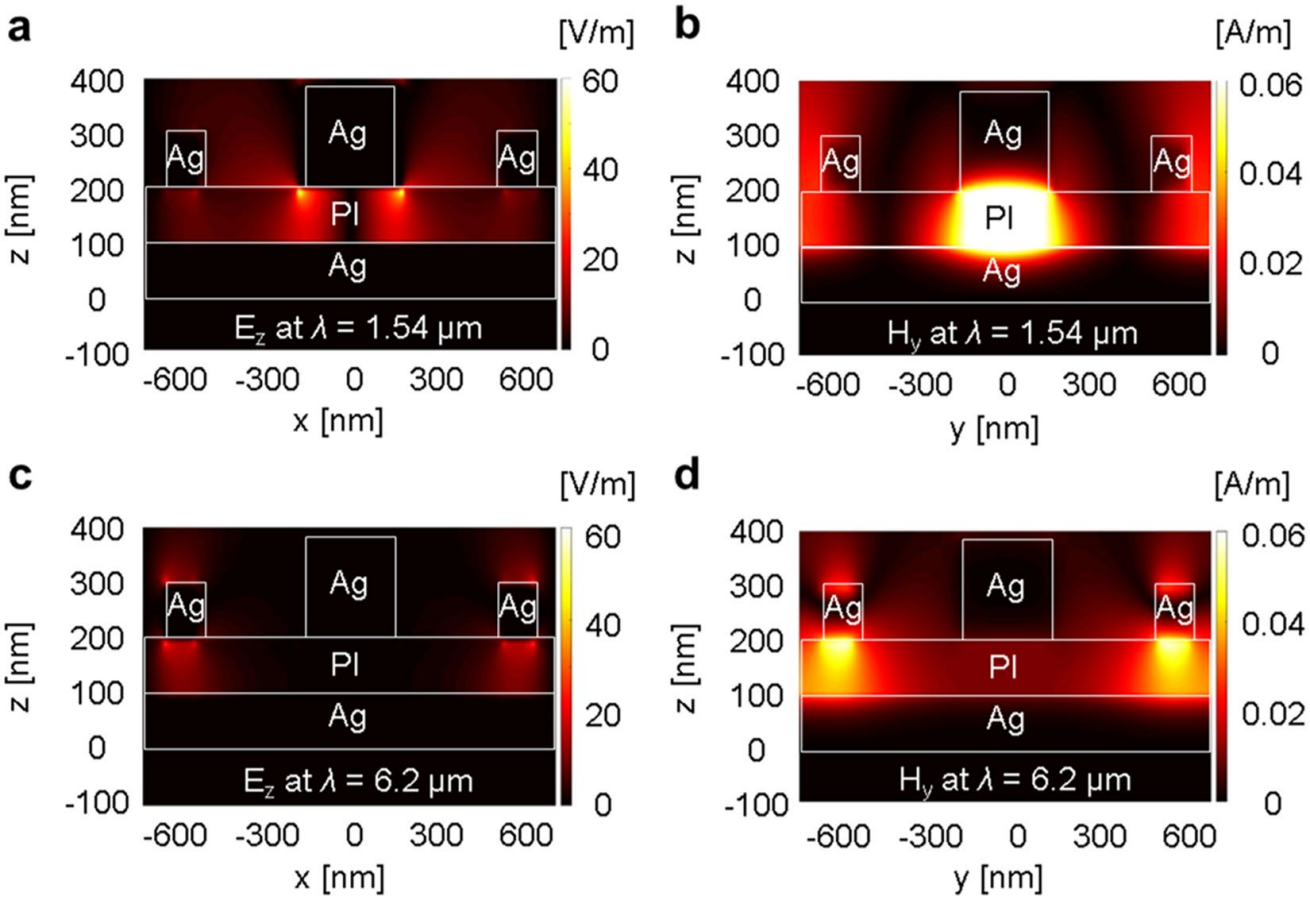
Electric and magnetic field distributions. (**a**) Electric field distribution E_x_ and (**b**) magnetic field distribution H_y_ in cross sections of the MIM structure when SPPs and MPs were simultaneously excited by the disk array at a wavelength of 1.54 μm. (**c**) E_x_ and (**d**) H_y_ in cross-sections of the MIM structure when MPs were excited only by the rings at a wavelength of 6.2 μm.

We calculated the electromagnetic field distributions in the cross section of the MIM structure at the SPP Bloch wave and MP resonance wavelengths by performing an FDTD simulation, whose results are shown in Fig. 3(a–d). In the simulation, the incident electric field E was normalized to 1 and the magnetic field was expressed as H = 1/Z_0_, where Z_0_ is the impedance of vacuum. At a resonance wavelength of 1.54 μm, the SPP Block waves excited by the disks were visualized using an electric field in the z-direction, as depicted in Fig. 3(a), which shows a strong electric field distribution due to the SPP resonance. At the same wavelength, a highly enhanced magnetic field in the y-direction was confined in the dielectric layer underneath each metal disk according to Lenz’s law^16^, as is evident from Fig. 3(b), which indicates that strong MPs were excited by the disks.

As shown in Fig. 2(a), we set the disk dimensions so that the SPP Bloch wave and MP resonances would occur at the same wavelength. Note that the conditions necessary to achieve the SPP Bloch wave and disk-generated MP resonances are still valid without the interference of the rings in the MIM structure. Therefore, we calculated the wavelengths of the resonances generated by the disks and rings separately using the ICCM and FDTD simulation, and combined the results of the two components to obtain the total response of the MIM structure.

Similarly, we calculated the electromagnetic field distribution at the wavelength of the ring-excited MP resonance. The electric and magnetic field distributions for the resonance wavelength of 6.2 μm are shown in Fig. 3(c) and (d), respectively. Since the rings in the MIM structure excite only the strong MPs at that wavelength, the FDTD simulation results exhibit a very weak electric field distribution underneath the ring in Fig. 3(c); however, a very strong magnetic field was excited by the ring because of the MP resonance, as shown in Fig. 3(d).

### Spectral properties of the dual-band metamaterial perfect absorber

**Figure 4:**
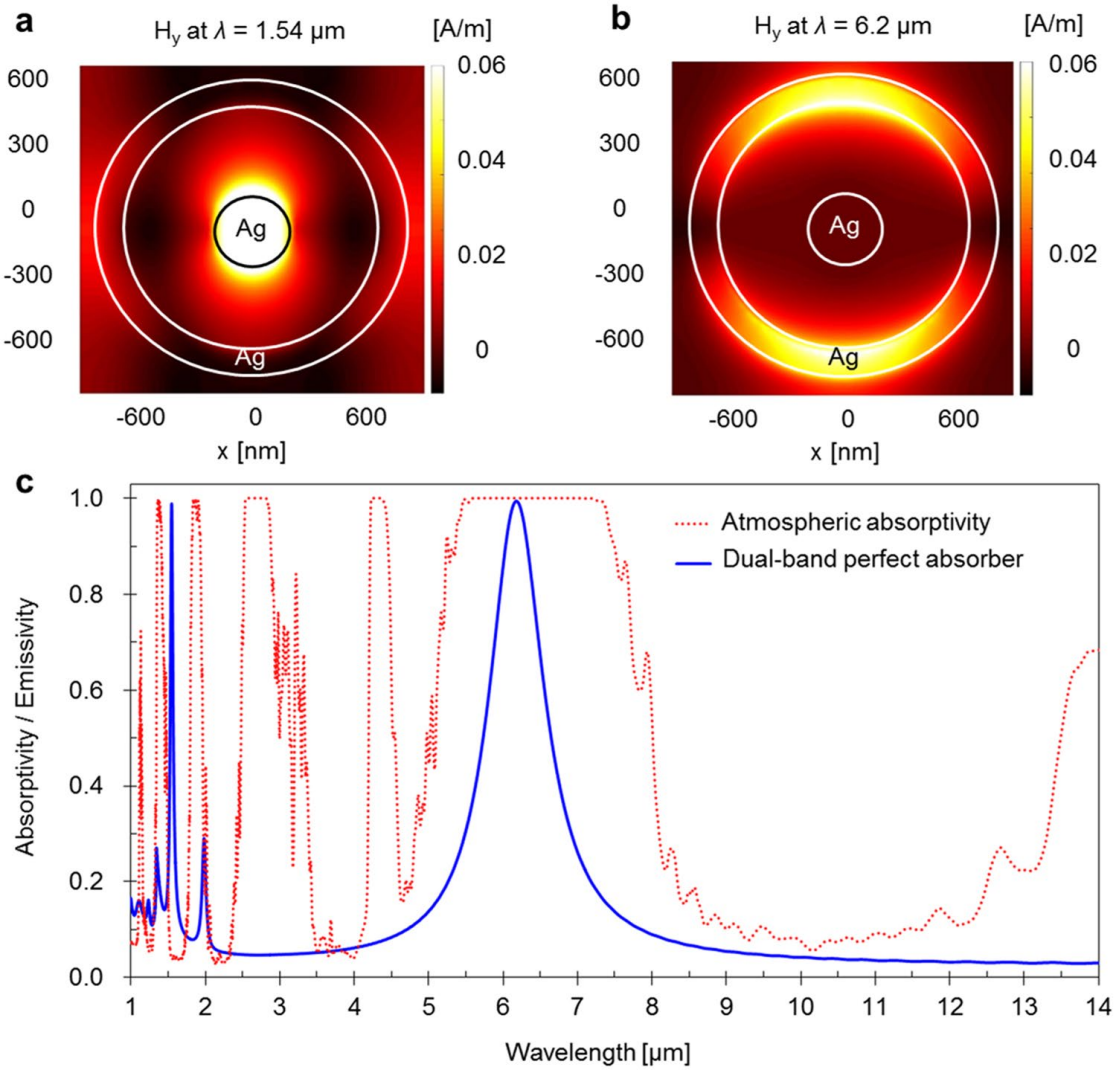
Magnetic field distributions and spectral properties. (**a**) and (**b**) Top views of the magnetic field distributions of the MPs calculated at 1.54 μm and 6.2 μm, respectively. (**c**) Spectral properties of the dual-band perfect absorber proposed in this work (solid line) and the atmospheric absorption spectrum (dotted line).

The magnetic field distributions of the MPs calculated at resonance wavelengths of 1.54 μm and 6.2 μm are shown in Fig. 4(a) and (b), respectively. The spectral properties of the dual-band perfect absorber proposed in this work are plotted in Fig. 4(c) with a solid line, while the atmospheric absorption spectrum is represented by the dotted line. The top views of the magnetic field distributions in the x-y plane of the MIM structure exhibit MP excitations around the disk and ring at 1.54 μm and 6.2 μm, respectively. Thus, the MPs are independently excited by the disks and rings at the two resonance wavelengths.

At 1.54 μm, the MPs interfere with the SPP Bloch waves, yielding a very narrow absorption peak due to the multiple interference of the SPPs generated by the unit cells periodically arranged in the 2D grating structure. By combining the resonance peaks of the SPP Bloch waves and MPs, a perfect absorber with an absorptivity of 99% can be realized at 1.54 μm. As shown in Fig. 4(c), the resonance peak at 1.54 μm has a very narrow full-width at half-maximum (FWHM) of 40 nm, which is important in IR stealth technology, because it can suppress any possible SWIR signatures.

We set the dimensions of the rings in the MIM structure to generate another MP resonance peak at 6.2 μm, which is in the middle of the strong absorption band of water in the atmosphere. As is evident from Fig. 4(c), the dual-band perfect absorber has a very low emissivity in the LWIR wavelength range (8–14 μm), enabling the reduction of thermal radiation that could be detected by many IR-tracking devices and imaging instruments.

We analysed the dependence of the spectral properties of the MIM structure on the polarization of the incident beam. Since the unit cells of the MIM structure have a circular symmetry, the resonance wavelengths of 1.54 μm and 6.2 μm are insensitive to polarization variations. Furthermore, the wavelength shifts of the 1.54 μm and 6.2 μm resonance peaks with polarization angle variations were found to be negligible compared with their bandwidths. The FWHMs of the 1.54 μm and 6.2 μm peaks were determined to be 40 nm and 1 μm, respectively.

**Figure 5:**
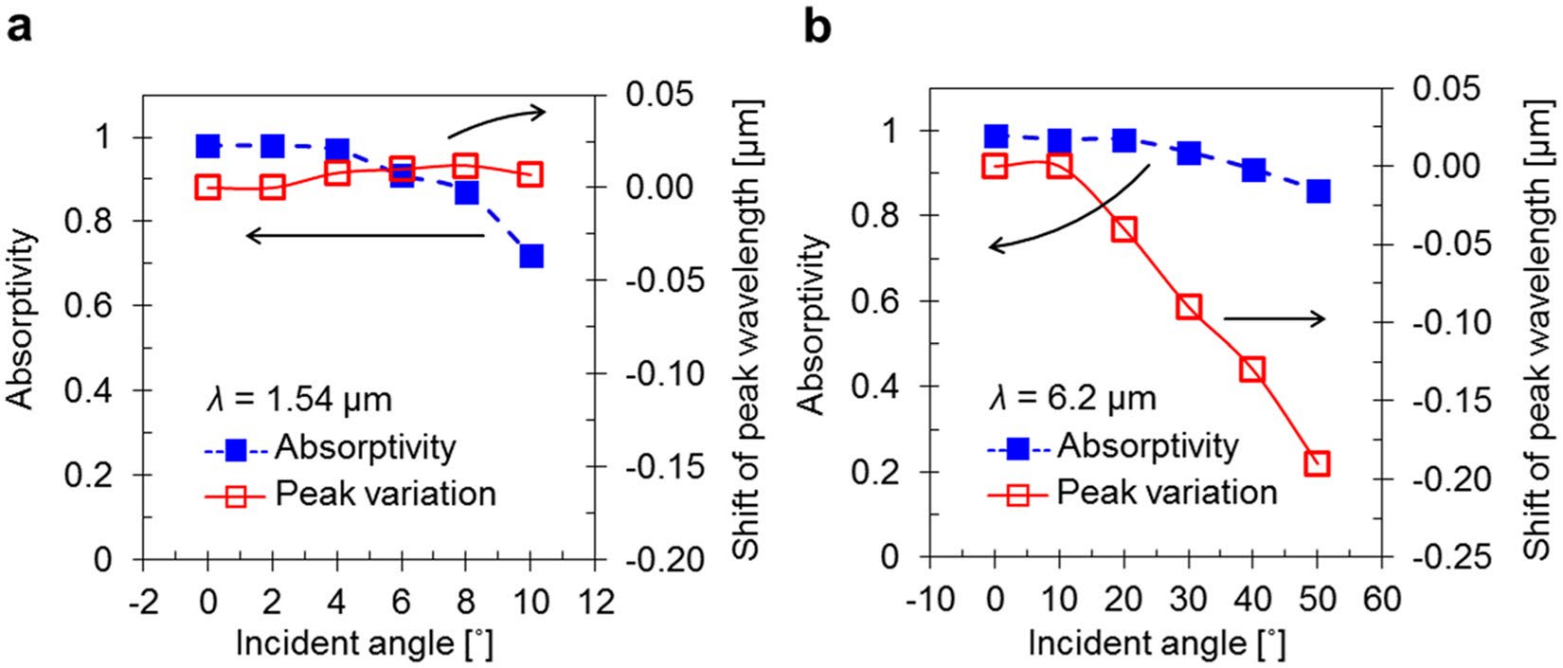
Incident angle sensitivity. Calculated absorptivities and wavelength shifts of the (**a**) 1.54 μm and (**b**) 6.2 μm MP peaks for various incident angles.

We also investigated the dependence of the absorptivity and resonance wavelength on the incident angle, and the results are shown in Fig. 5(a) and (b) for the 1.54 μm and 6.2 μm resonance peaks, respectively. We tuned the resonance peaks of the SPP Bloch waves and MPs so that they both occurred at 1.54 μm. As shown in Fig. 5(a), we analysed the shifts of the 1.54 μm MP resonance peak at incident angles less than 10°, since laser-guided missiles detect light scattered from target surfaces within small collection angles. The resonance wavelength remains fixed at 1.54 μm with small variations of 40 nm, even though the magnitude of the peak gradually decreases by 25% when the incident angle varies by 10°. Compared to the wavelength of the 1.54 μm resonance peak, that of the 6.2 μm MP resonance peak is slightly more sensitive to incident angle variations; however, the absorptivity at the MP peak is almost insensitive to incident angle variations up to 50°. We can conclude that the absorptivity at different incident angles is related to the emissivity of thermal radiation by the surface that is detected at the incident angle. Therefore, the MIM structure causes the absorber to remain a perfect absorber across a wide range of detection angles.

### Usability of the dual-band metamaterial perfect absorber in IR stealth technology

**Figure 6:**
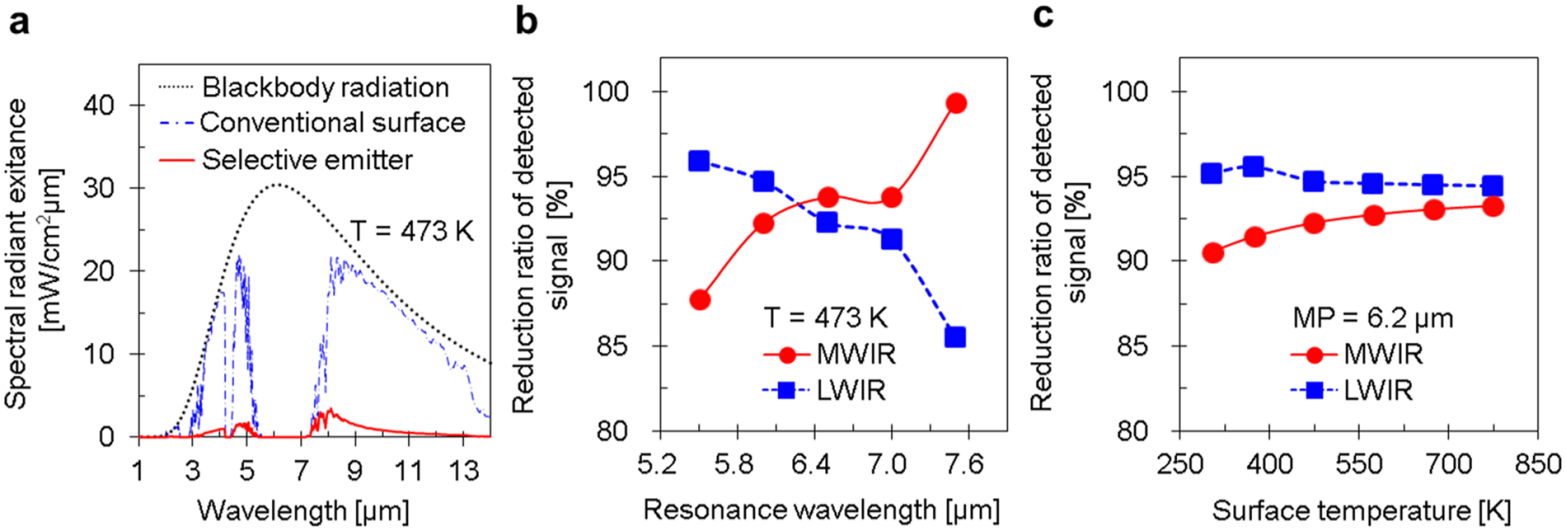
Usability of the dual-band metamaterial perfect absorber in IR stealth technology. (**a**) Spectral radiant exitances of a blackbody (calculated using Planck’s radiation law at 473 K (dotted line)), a conventional surface (dashed-dotted line), and the perfect absorber (solid line), assuming a 1-km-long transmission path through the atmosphere at ground level. (**b**) Reduction rates of IR signatures in the MWIR and LWIR wavelength ranges (3–5.5 μm and 8–14 μm, respectively) for various wavelengths of the ring-excited MP resonance. The reduction rates were determined by comparing the spectral radiances emitted by the perfect absorber and a blackbody, which were transmitted through the atmosphere. (**c**) The same ratios as in (**b**) calculated for various surface temperatures with a resonance wavelength of 6.2 μm.

We evaluated the usability of the proposed absorber in IR stealth technology in two wavelength ranges—the MWIR wavelength range from 3 μm to 5.5 μm and the LWIR wavelength range from 8 μm to 14 μm—since IRST detectors employ the signatures of objects in these wavelength ranges. The atmospheric absorption band was obtained using a commercial software—MODTRAN (MODerate-resolution atmospheric TRANsmission). In the calculations, we assumed that the IR signature from the object was defined by the thermal radiation transmitted at a horizontal distance of 1 km through the atmosphere at ground level. In Fig. 6(a), the spectral radiant exitance of the blackbody radiation at 473 K is compared with the calculated IR signatures of a blackbody and the perfect absorber. The IR signatures in the MWIR and LWIR ranges can be easily derived by integrating the spectral radiant exitance in these wavelength ranges^35^. Figure 6(a) shows that the IR signatures in the MWIR and LWIR wavelength ranges can be reduced by 92.1% and 92.6%, respectively.

To evaluate the usability of the perfect absorber in IR stealth technology, we calculated the reduction rate of the IR signature of blackbody radiation by using the perfect absorber. The reduction rates calculated for various wavelengths of the ring-excited MP resonance are shown in Fig. 6(b). Owing to the tails of the peak that are evident in Fig. 4, the reduction rates exhibit opposite trends in the MWIR and LWIR wavelength ranges. As the resonance wavelength increases, the IR signature of the perfect absorber decreases in the MWIR range and increases in the LWIR range. Nonetheless, the perfect absorber designed in this work exhibits good performance in the MWIR and LWIR wavelength ranges simultaneously. We also calculated the reduction rates at various temperatures, which are plotted in Fig. 6(c). The reduction rates of the IR signature in the MWIR and LWIR ranges change slightly, exhibiting opposite trends. As the temperature increases, the IR signature in the MWIR range increases, while that in the LWIR range decreases. Notably, the reduction rate is greater than 90% across a wide temperature range from room temperature to 500 °C.

### Consideration of design parameters for manufacturing

For the fabrication of the double-band perfect absorber proposed in this work, a double lithography step was required due to the different thicknesses of the disk and the ring. To reduce the manufacturing complexity, we designed the perfect absorber such that it could be fabricated using a single lithography step. For comparison, the design parameters for manufacturing using different lithography processes are listed in Table 1. Considering the independent resonance characteristics of the circular ring and disk, we fixed the design parameters of the ring for the MP2 peak and fixed the dimensions of the disk such that the thicknesses t_2_ and t_1_ were the same (100 nm).

**Table 1:** Design parameters of the perfect absorber for different lithography methods.

Parameters [nm]	*d*	*t*_1_	*t*_2_	*r*_1_	*r*_2_	*r*_3_
Fabrication						
Single lithography step	0.100	0.100	0.100	0.175	0.500	0.600
Double lithography step	0.100	0.100	0.180	0.168	0.500	0.600

The period of disk *a*_0_ is set to 1.34 μm for both the designs.

To fabricate the dual-band perfect absorber using a single lithography step, we analysed the usability of the IR stealth technology. The results are described in detail in the Supplementary Information. In terms of the availability of the dual-band perfect absorber in IR stealth technology, the most important difference between the two designs is the incident angle dependence of absorption in the 1.54 um band. For an absorption greater than 75%, the perfect absorber using the double lithography step allows a range of 9°, while that using the single lithography step allows a much narrower range for the incident angle (less than 5°). For the laser-guided missile, in which the receiver and detectors are combined, the dependence of the performance of the IR stealth technology on the incident angle is negligible^23^. However, if the detector is separated from the receiver, the absorptivity at a wide incident angle may be considered as a significant performance^23^. Fortunately, however, except for the incident angle dependence in the 1.54 μm band, both the designs exhibit almost identical performances.

## Discussion

We have proposed a dual-band metamaterial perfect absorber for use in IR stealth technology. To realize the perfect absorber, we designed an MIM structure with unit cells yielding SPP and MP resonance peaks at 1.54 μm and 6.2 μm, respectively. The perfect absorber has a narrow IR absorption band at 1.54 μm to suppress the scattering signals used by laser-guided missiles and a broad thermal radiation band at 6.2 μm to reduce the MWIR and LWIR signatures by employing atmospheric absorption. We minimized the wavelength shift of the narrow IR absorption peak at 1.54 μm via variations in the incident angle of light by exciting SPPs using the Bloch-wave condition and the MPs of the disks simultaneously, while maintaining a high absorption peak. By utilizing the MPs excited by the rings at 6.2 μm, the absorber can remain a perfect absorber with negligible resonance wavelength shifts at various incident angles.

We analysed the spectral properties of the resonance peaks at 1.54 μm and 6.2 μm by comparing the MP peak wavelength calculated based on the FDTD simulation results with that obtained using an ICCM. The wavelength shift of the 1.54 μm MP peak was found to be less than 10 nm for incident angle variations of 10°, while that of the 6.2 μm MP peak was less than 200 μm for incident angle variations of 50°. We also evaluated the usability of the dual-band metamaterial perfect absorber in IR stealth technology. The absorber was able to reduce the scattering of a 1.54 μm IR laser light by more than 90%. Furthermore, the MWIR and LWIR signatures could be suppressed by more than 92% using the dual-band perfect absorber and were reduced by at least 90% across a wide temperature range, from room temperature to 500 °C.

In addition, to avoid manufacturing complexity due to the double lithography step, we designed the perfect absorber such that it could be fabricated using a single lithography step. The performance of the perfect absorber, which can be fabricated using a single lithography step, has a smaller allowance range for the incident angle in the 1.54 µm band compared to that using the double lithography step. It was found that except for the incident angle dependence in the 1.54 μm band, both the designs exhibit almost the identical performances.

## Methods

### Numerical simulation

We employed an FDTD method (using Lumerical FDTD Solutions software) to verify the modelling process, ICCM, and SPP Bloch conditions and to evaluate the performance of the selective dual-band structure. Periodic boundary conditions were applied in the x and y directions of the unit cell in the normal incidence case, and Bloch boundary conditions were adopted when the incident angle was varied. Along the z-direction, perfectly matched layers were used. The incident light consisted of TM-mode plane waves. A nonuniform mesh with a minimum mesh size of 5 nm was employed.

## Electronic supplementary material

Supplementary Information 
