# Supplementary material for: Selective dual-band metamaterial perfect absorber for infrared stealth technology
Source: Sci Rep. 2017 Jul 27;7:6740. doi: 10.1038/s41598-017-06749-0 (PMC5532238; doi:10.1038/s41598-017-06749-0)
Supplement: Supplementary file 1 — Supplementary Information [file 41598_2017_6749_MOESM1_ESM.pdf]

Supplementary Information

Selective dual-band metamaterial perfect absorber for infrared stealth technology

Jagyeong Kim, Kiwook Han, and Jae W. Hahn*

Nano Photonics Laboratory, School of Mechanical Engineering, Yonsei University,

50 Yonsei-ro, Seodeamun-gu, 03722 Seoul, Republic of Korea

*Corresponding author: jaewhahn@yonsei.ac.kr

Inductor-capacitor circuit model for disks and rings in a metal–insulator–metal structure

When incident light encounters a disk in a metal–insulator–metal (MIM) structure, the interaction between the disk and the light can be represented using the equivalent inductor–capacitor circuit (ICC), depicted in Fig. S1[1]. The neighbouring unit cells are represented by the gap capacitances in parallel, and within each unit cell, there is a series circuit, which includes mutual inductances, kinetic inductances, and mutual capacitances. The impedance of the ICC for a circular disk becomes zero at resonance, and is represented by the following equation[1]:

$$Z = \frac{i\omega\left(L_{\mathrm{m1}} + L_{\mathrm{k1}}\right)}{1 - \omega^2 C_{\mathrm{g1}}\left(L_{\mathrm{m1}} + L_{\mathrm{k1}}\right)} - \frac{2i}{\omega C_{\mathrm{m1}}} + i\omega\left(L_{\mathrm{m1}} + L_{\mathrm{k1}}\right), \qquad \text{(S1)}$$

where the parameters are defined in Tables S1 and S2.

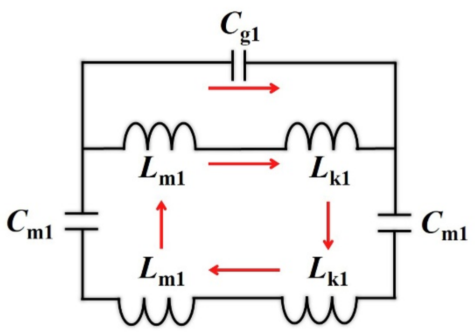

Figure S1. Equivalent ICC of a disk in an MIM structure[1].

Geometrical parameter or material property	*Description*
a_0	Period of a unit cell in the circular disk–ring metamaterial
d	Thickness of the dielectric layer
t_1 and t_2	Thicknesses of the circular disks and metal rings, respectively
r_1	Radius of the circular disks
r_2 and r_3	Inner and outer radii, respectively of the circular rings
ε_0 and ε_{PI}	Permittivities of free space and dielectric layer (polyimide), respectively
ε' and ε''	Real and imaginary parts, respectively, of the permittivity of the metal layer (silver, Ag)
δ	Penetration depth
μ_0	Permeability of free space

Table S1. Geometrical parameters and material properties for the circular ring and disk structure.

ICC parameter	*Description*
c_{disk}	Non-uniform charge distribution coefficient, which was determined to be 0.08 in this study by performing a finite-difference time-domain (FDTD) simulation for the circular disk structure
l_{disk}	Length of the surface current on a circular disk, $l_{\text{disk}} = 2\pi r_1$
$A_{1,\text{disk}}$	Area of a circular disk encountering the lower layer, $A_{1,\text{disk}} = \pi r_1^2$
C_{m1}	Mutual capacitance, $C_{\text{m1}} = c_{\text{disk}}\varepsilon_{\text{PI}}\varepsilon_0 A_{1,\text{disk}} / d$
C_{g1}	Gap capacitance, $C_{\text{g1}} = c_{\text{disk}}\varepsilon_0 A_{2,\text{disk}} / (a_0 - 2r)$
L_{m1}	Mutual inductance, $L_{\text{m1}} = 0.5\mu_0 l_{\text{disk}} d / 2r_1$
L_{k1}	Kinetic inductance, $L_{\text{k1}} = \left(-1/\varepsilon_0\omega^2\delta\right)\left(\varepsilon' / \left(\varepsilon'^2 + \varepsilon''^2\right)\right)$

Table S2. Parameters in the ICC for the circular disk structure.

Similar to the interaction between the incident light and a circular disk, the interaction between the incident light and a circular ring in the MIM structure can be represented using the ICC illustrated in Fig. S2(a). The periodic unit cells are related to one another in parallel via gap capacitances, and within each unit cell, there is a series circuit, which includes mutual inductances, kinetic inductances, and mutual capacitances.

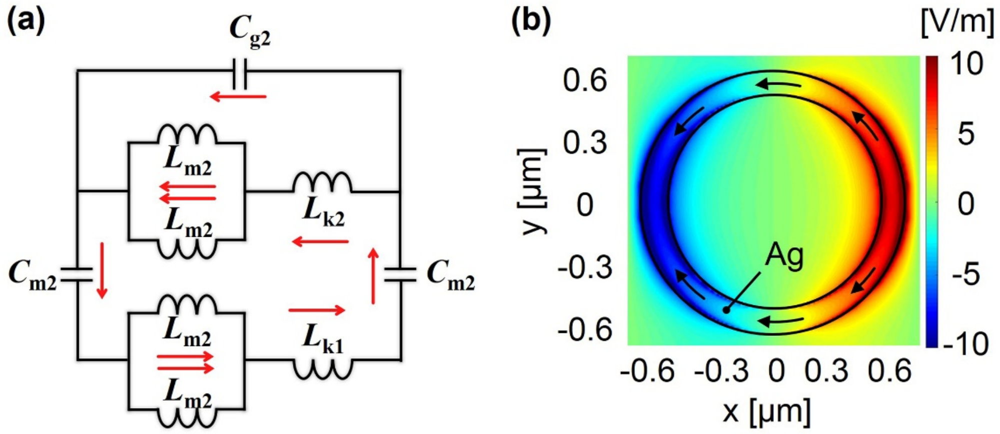

Figure S2. (a) Equivalent ICC for a ring in a unit cell of the MIM structure. (b) Electric field E_z calculated using the FDTD method, representing the distribution of opposite charges on the upper side of the circular ring. The current flow is indicated with the series of arrows.

When incoming light is incident on the absorber, an odd-symmetric charge distribution is created on the upper side of the ring, as shown in Fig. S2(b). As illustrated in Fig. S2(a), two currents following in opposite directions along the ring are induced in the upper metal layer of the ring. It can be expected that two parallel currents flowing in opposite directions would be simultaneously induced in the lower metal layer, forming current loops. According to Lenz's law, a magnetic field is confined within the dielectric space between the current flows in two metal layers[2]. Therefore, mutual inductance is created between the lower and upper metal layers.

Due to the vibrations of the free electrons, caused by the currents on the metal surfaces, kinetic inductances are generated on the lower and upper metal plates. As they depend upon the shapes of the surfaces in which the currents flow, the kinetic and mutual inductances are related in series, as shown in Fig. S2(a). Unlike the mutual inductance in the lower plate, which depends only on whether the symmetrical currents flow in a direction opposite to the current in the circular ring, the kinetic inductance in the lower layer is induced throughout the area in which the current flows. Therefore, the characteristics of the kinetic inductance on the lower plate are identical to those in the circular disk array, while those of the kinetic inductance on the upper metal plate are different, due to the air space in the circular ring geometry that is related to the inner radius. To perform resonance wavelength predictions, we derived the total impedance of the ICC for the circular ring shown in Fig. S2(a), which can be expressed as[3]

$$Z = \frac{i\omega\left(L_{\mathrm{m2}} + 2L_{\mathrm{k2}}\right)}{2 - \omega^2\left(L_{\mathrm{m2}} + 2L_{\mathrm{k2}}\right)C_{\mathrm{g2}}} - \frac{2i}{\omega C_{\mathrm{m2}}} + i\omega\left(\frac{L_{\mathrm{m2}}}{2} + L_{\mathrm{k1}}\right), \tag{S2}$$

where the parameters are defined in Table S3. When the impedance is zero, like that of free space, the incident light is almost perfectly absorbed by the circular ring structure. We set the outer radius of the circular rings to 600 nm and the unit cell period to 1.34 μm. Finally, we determined that the inner radius is 500 nm when the magnetic polariton resonance wavelength is 6.2 μm.

ICC parameter	***Description***
c_{ring}	Non-uniform charge distribution coefficient, which was determined to be 0.08 in this study by performing an FDTD simulation of the circular ring structure
l_{ring}	Length of the surface current on a circular ring, $l_{\text{ring}} = \pi\left(r_3 + r_2\right)/2$
$A_{1,\text{ring}}$	Surface area of a circular ring encountering the lower layer, $A_{1,\text{ring}} = \pi\left(r_3^{\,2} - r_2^{\,2}\right)$
$A_{2,\text{ring}}$	Surface area of a circular ring facing a neighbouring unit cell, $A_{2,\text{ring}} = \pi r_3 t_2$
C_{m2}	Mutual capacitance, $C_{\text{m2}} = c_{\text{ring}}\varepsilon_0\varepsilon_{\text{PI}}A_{1,\text{ring}}/d$
C_{g2}	Gap capacitance, $C_{g2} = c_{\text{ring}}\varepsilon_0 A_{2,\text{ring}}/\left(a_0 - 2r_3\right)$
L_{m2}	Mutual inductance, $L_{\text{m2}} = 0.5\mu_0 l_{\text{ring}} d/\left(r_3 - r_2\right)$
L_{k1}	Kinetic inductance at the surface of the lower metal layer, $L_{\text{k1}} = \left(-1/\varepsilon_0\omega^2\delta\right)\left(\varepsilon'/\left(\varepsilon'^2 + \varepsilon''^2\right)\right)$
L_{k2}	Kinetic inductance at the surface of the upper metal layer, $L_{k2} = \left(-1/\varepsilon_0\omega^2\delta\right)\left(\varepsilon'/\left(\varepsilon'^2 + \varepsilon''^2\right)\right)\left(2l_{\text{ring}}/\left(r_3 - r_2\right)\right)$

Table S3. Parameters in the ICC for the circular ring structure.

Usability of the perfect absorber to be fabricated using single lithography step

For the fabrication process using single lithography step, we additionally designed a dual-band metamaterial absorber with the same thicknesses for the metal disk and ring. Considering the independent resonance characteristics of the circular ring and disk, we fixed the design parameters of the ring for the MP2 peak and fixed the dimensions of the disk such that the thicknesses t_1 and t_2 were the same (100 nm). The spectral properties of the dual-band perfect absorber designed for the single lithography step (solid line) is displayed along with the atmospheric absorption spectrum (dotted line) in Fig. S3. On comparing the wide spectral property in Fig. S3 with that in Fig. 4, we found no significant difference in the spectral properties of the two perfect absorbers designed for double lithography step and single lithography step.

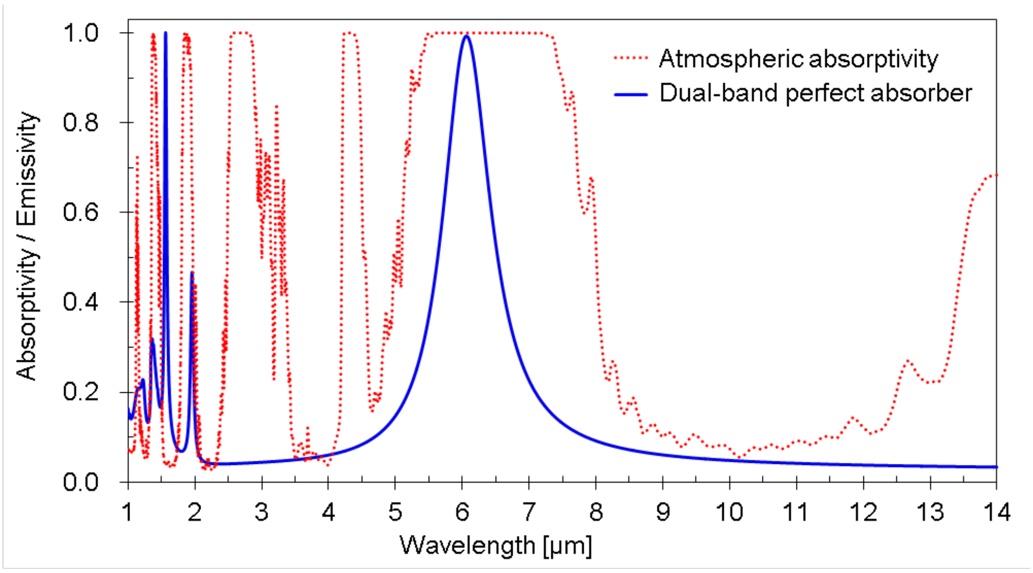

Figure S3. Spectral properties of the dual-band perfect absorber designed for single lithography step (solid line) is displayed along with the atmospheric absorption spectrum (dotted line).

When the various spectral properties of the perfect absorber, designed for single lithography step, was investigated closely, it was found that the absorptivity at 1.54 μm is much sensitive to the variations in incident angle. The dependences of absorptivity and resonance wavelength on the incident angle are plotted in Figs. S4 (a) and (b) for the peaks of 1.54 μm and 6.2 μm, respectively. It is noticeable that the absorptivity of the 1.54 μm resonance peak is very sensitive and sharply decays as a function of the incident angle. Compared with that for the perfect absorber designed for double lithography step, the decay rate in Fig. S4 (a) is 1.8 times larger than that in Fig. 5 (a). For the laser guided missile, in which the receiver and detectors are combined, the performance of IR stealth technology depending on the sensitivity of incident angle is negligible[23]. However, if the detector is separated from the receiver, the absorptivity at a wide incident angle may be considered as an important performance [23]. Fortunately, however, except for the incident angle dependence in the 1.54 μm band, both the designs exhibit almost the same performances.

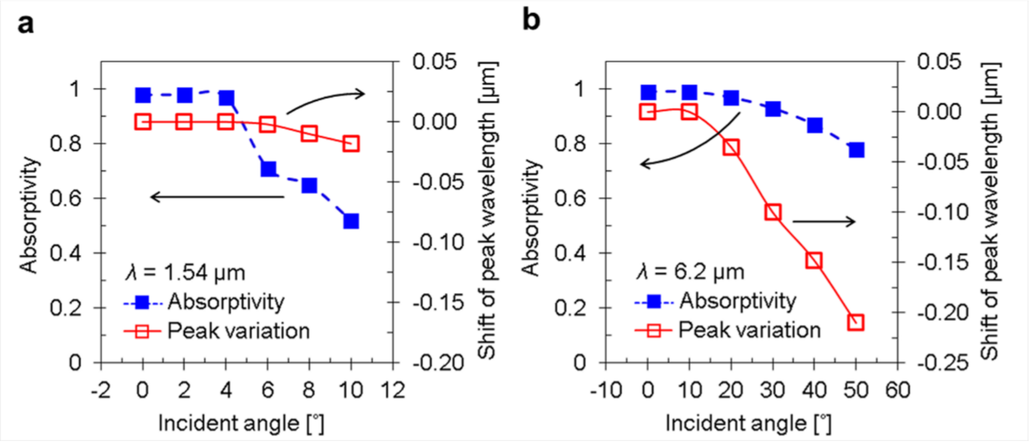

Figure S4. Calculated absorptivities and wavelength shifts of the perfect absorber designed for single lithography step for various incident angles. The results are plotted for the MP resonance peak at (a) 1.54 μm and (b) 6.2 μm.

References

1. Atsushi Sakurai, Bo Zhao, Zhuomin M. Zhang, Proceedings of the 15th International Heat Transfer Conference, IHTC-15, (2014)

2. L. P. Wang, Z. M. Zhang, “Wavelength-selective and diffuse emitter enhanced by magnetic polaritons for thermophotovoltaics”, APL, 100, 121116, (2012)

3. Foundations of electromagnetic theory, 4th edition, John.R.Reitz et al. Addison-Wesley, (2008)
